# The long‐term safety of topical corticosteroids in atopic dermatitis: A systematic review

**DOI:** 10.1002/ski2.268

**Published:** 2023-08-16

**Authors:** Jane Harvey, Stephanie J. Lax, Alison Lowe, Miriam Santer, Sandra Lawton, Sinead M. Langan, Amanda Roberts, Beth Stuart, Hywel C. Williams, Kim S. Thomas

**Affiliations:** ^1^ Centre of Evidence Based Dermatology School of Medicine University of Nottingham Nottingham UK; ^2^ University Hospitals Sussex NHS Foundation Trust Worthing UK; ^3^ Primary Care Research Centre University of Southampton Southampton UK; ^4^ Department of Dermatology Rotherham NHS Foundation Trust Rotherham UK; ^5^ London School of Hygiene and Tropical Medicine London UK; ^6^ Nottingham Support Group for Carers of Children with Eczema Nottingham UK; ^7^ Wolfson Institute of Population Health Queen Mary University of London London UK

## Abstract

**Background:**

Topical corticosteroids (TCS) are a first‐line treatment for eczema, but there are concerns about their safety when used long‐term.

**Objectives:**

To systematically review adverse effects associated with longer‐term use of TCS for eczema.

**Methods:**

Randomised controlled trials (RCTs), cohort and case‐control studies reporting adverse effects of TCS (comparators: no TCS treatment, other topicals) in patients with eczema were identified. Included studies had greater than one year of follow‐up, minimum cohort size of 50 participants, or minimum 50 per arm for RCTs. Evidence was GRADE‐assessed. Prospero registration CRD42021286413.

**Results:**

We found seven studies (two randomised, five observational); two RCTs (*n* = 2570, including 1288 receiving TCS), two cohort (all received TCS *n* = 148) and three case‐control studies (cases *n* = 10 322, controls *n* = 12 201). Evidence from two RCTS (*n* = 2570, children, three and five years' duration) comparing TCS to topical calcineurin inhibitors found intermittent TCS use probably results in little to no difference in risk of growth abnormalities, non‐skin infections, impaired vaccine response and lymphoma/non lymphoma malignancies. The five‐year RCT reported only one episode of skin atrophy (*n* = 1213 TCS arm; mild/moderate potency), suggesting TCS use probably results in little to no difference in skin thinning when used intermittently to treat flares. No cases of clinical adrenal insufficiency were reported in 75 patients using mild/moderate TCS in the three‐year RCT. Small associations between TCS and type‐2 diabetes and lymphoma were identified in two case‐control studies compared to no TCS, but the evidence is very uncertain. No long‐term studies concerning topical steroid withdrawal or eye problems were identified.

**Conclusion:**

This review provides some reassuring data on growth and skin thinning when TCS are used intermittently for up to 5 years, but many knowledge gaps remain.

## Graphical abstract

This is a systematic review of the long‐term safety of topical corticosteroids for the treatment of atopic dermatitis. The review includes GRADE assessments of the evidence from both randomised controlled trials and observational studies (cohort and case‐control studies) with greater than one year of follow‐up.

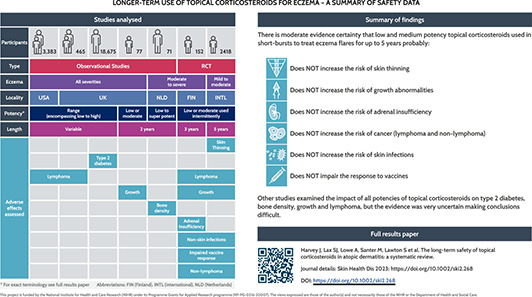

**What is already known about this topic?**

- Patients, carers and health professionals are often concerned about potential adverse effects of using topical corticosteroids (TCS). Such concerns can result in under‐treatment of eczema, resulting in poor quality of life.
- Information from relevant studies of longer‐term safety may help patients, carers and health professionals make informed decisions about TCS treatment.
- Recent systematic reviews of TCS safety are restricted in scope or require updating.

**What does this study add?**

- This review summarised the existing evidence for the safety of TCS when used for more than a year. It includes randomised controlled trials (RCTs) and observational studies.
- Overall, the limited body of evidence reviewed provided some indication that TCS used intermittently for the management of eczema is safe over periods of up to 5 years.
- Better quality studies which address all relevant safety outcomes and include longer follow‐up are needed.

## 1 INTRODUCTION

Eczema (also called ‘atopic dermatitis’ (AD) or ‘atopic eczema’^1^) is a common chronic, condition characterised by dry itchy skin. It is probably a heterogeneous condition.^2^ Although around 80% of children with eczema appear to outgrow their condition, many continue to suffer into adulthood,^3^ resulting in use of eczema medications for many years. Topical corticosteroids are commonly prescribed to people with eczema and are often used intermittently along with emollients as a first line treatment over the course of a lifetime for those with persistent disease.^4^

Eczema itself has been linked to poor health outcomes, including reduced quality of life,^5^ as well as increased risk of fractures,^6^ lymphoma^7^ and cardiovascular disease.^8^

An adverse effect is defined as “an unfavourable outcome that occurs during or after the use of a drug or other intervention and the causal relation between the intervention and the event is at least a reasonable possibility”.^9^ Patients often worry about the long‐term adverse effects of TCS, and the long‐term safety of TCS has been prioritised for future research.^10^

To evaluate the long‐term safety of treatments, it is necessary to evaluate observational studies that cover a longer timeframe than is realistically possible in most randomised controlled trials (RCTs). Recent systematic reviews of TCS safety which include observational studies are restricted to specific adverse effects, include only one type of drug or strategy of using one type of drug, or require updating.^11^ This systematic review includes both RCTs and observational studies and summarises the available evidence on known long‐term adverse effect of TCS to help patients, carers and health professionals make informed decisions about eczema management.

## 2 METHODS

### 2.1 Protocol registration

The protocol for this review was registered on PROSPERO on 5/11/2021, Registration Number CRD42021286413.

### 2.2 Differences with the protocol

We reduced the number of participants per study from 100 to 50 to be more inclusive, and we clarified that quality assessments for case‐control studies would be done using the Newcastle Ottawa Scale rather than the ROBINS‐I tool (See Supplementary Appendix S1).

### 2.3 Inclusion criteria for this systematic review

#### 2.3.1 Types of study

Randomised controlled trials, cohort studies with a comparator group and case control studies. All studies had greater than one year of follow‐up and a minimum cohort size of 50, or minimum 50 per group for RCTs. We required studies to have these sample sizes to have sufficient precision to estimate effects.

#### 2.3.2 Types of participants

People with eczema, any age, any sex, from any setting. We included only studies in patients with ad in order to specifically address the research question “What is the long term safety of applying steroids to the skin for eczema?” highlighted by the James Lind Alliance Eczema Priority Setting Partnership.^10^ Furthermore, we only included patients with ad due to the variation in the signs and symptoms associated with different skin diseases. For example, patients with vitiligo would not usually be applying TCS to broken skin, whereas application to broken skin is common in the treatment of AD.

#### 2.3.3 Types of interventions

Studies included TCS of any potency, preparation and regimen which were compared to either no TCS treatment or compared to other topical treatments.

#### 2.3.4 Types of outcomes

The main outcomes of interest were based on lists of key adverse effects identified in a previous systematic review of TCS safety.^12^ Further adverse effects were added after discussions within the author team which included two dermatologists, two consultant nurse specialists in dermatology and a GP. Two patients (AR and AA) also contributed to the development of this list. These adverse effects were discussed and through consensus we decided upon which adverse effects were “long‐term”. Another dermatologist (JR), independent of the author team, verified these decisions.

Pre‐specified adverse effects of interest were:

**Local adverse effects** – skin thinning, ageing, wrinkling, changes in skin colour, telangiectasia, worsening or induction of acne, striae and sensitisation that occurs after long‐term use.

We also looked for studies that concerned topical steroid withdrawal (TSW), identified from the terms described by Hajar 2015.^13^

**Systemic adverse effects** – bone problems such as osteoporosis and fractures, impact on growth, effects on endocrine system, eye problems, cancer, and mental health issues (anxiety, depression and attention deficit hyperactivity disorder).

We did not include local adverse effects that are associated with the immediate application of TCS for example, burning, stinging sensitivity, periocular dermatitis, application site reactions, skin infections, folliculitis, perioral dermatitis (not associated with withdrawal).

Although not listed above, we also extracted data on “non‐skin” based infections and vaccine response as it was recognised as an area of concern for patients.

### 2.4 Search methods

We searched MEDLINE via Ovid (from 1946 onwards) and Embase via Ovid (from 1974 onwards) up until 09/12/2021. This was using the search terms identified in Supplementary Appendix S2 developed in consultation with two information specialists (SB, DG).

In addition, we checked for RCTs included in two Cochrane reviews on topical treatments for eczema being conducted by the same authorship.^12^, ^14^ The database search from the Cochrane review “Topical anti‐inflammatory treatments for eczema: network meta‐analysis” was updated on 13/1/2023 with the clinical trial registry search conducted on 19/1/2023. Included studies within these reviews and within this review were hand searched to identify further trials.

### 2.5 Selection of studies

Two reviewers (either JH, SJL or AL) independently assessed titles and abstracts and subsequently full papers for relevance. Disagreements were reconciled between the reviewers or resolved by another reviewer (KST). No relevant foreign language studies were identified.

Where further information was required that was deemed essential for selection into the review or for further analysis of a particular study, we contacted study authors.

### 2.6 Data extraction and management

Title and abstract screening was completed using Rayyan (https://www.rayyan.ai/), full paper screening using Microsoft Excel. A PRISMA flow diagram was produced using the open access software produced by Haddaway *et al.* 2022.^15^ Two reviewers (either JH, SJL or AL) independently extracted data using a bespoke, piloted data extraction form in Microsoft Excel. Disagreements were reconciled between reviewers or resolved by a further reviewer (KST).

### 2.7 Data synthesis

Evidence was reported narratively, by adverse effect. All relevant data was extracted if reported at the furthest timepoint after 1 year.

### 2.8 Assessment of risk of bias

Quality of the studies was assessed by two independent reviewers (JH, SJL) using Cochrane RoB2^16^ for RCTs, ROBINS‐I tool^17^ for cohort studies and Newcastle Ottawa Case‐Control assessment tool^18^ for case‐control studies. A third reviewer resolved any conflicts (KST).

### 2.9 Assessment of confounders

A list of confounders, used in the ROBINS‐I and Newcastle Ottawa assessments, was pre‐specified through discussions within the author team and detailed within the protocol. Critical confounders included age, duration of eczema, and severity of eczema. In addition, we considered the effect of critical co‐interventions if systemic adverse effects were reported. These included all systemic corticosteroids (i.e., oral, inhaled, and parenteral corticosteroids).

### 2.10 Summary of findings and assessment of the certainty of the evidence

For this review, GRADE^19^ assessments for the evidence relating to each adverse effect were completed by two assessors (JH, SL) through dialogue and ratified by discussion with a third reviewer (KT) and a content expert (HCW). Separate GRADE assessments were conducted for observational studies and RCTs.

This review adheres to the PRISMA 2020 statement.^20^

## 3 RESULTS

### 3.1 Characteristics of included studies

**FIGURE 1:**
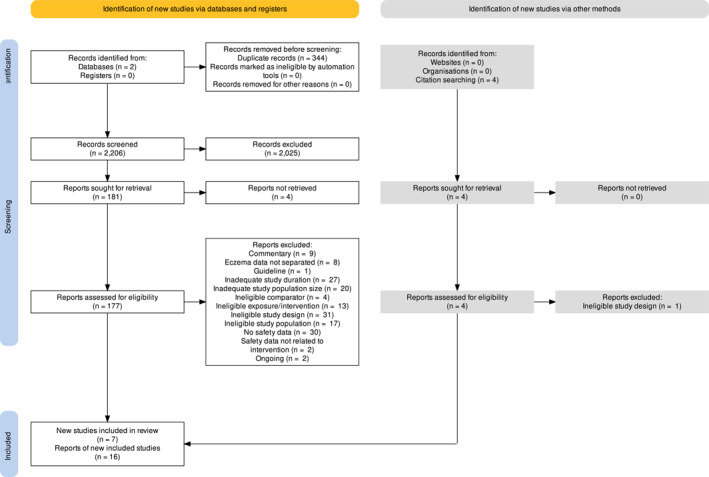
PRISMA flow diagram.

**FIGURE 2:**
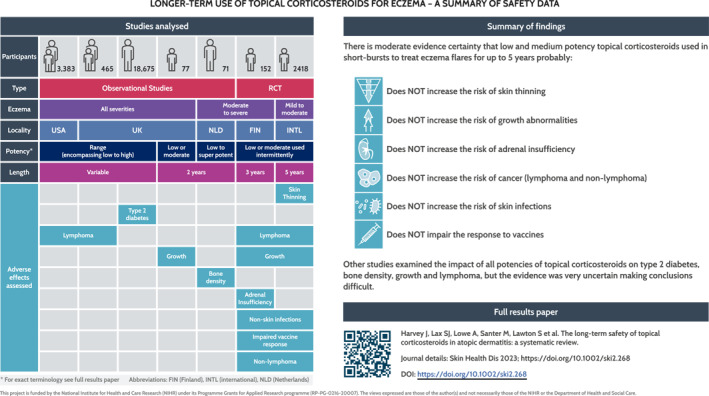
Infographic.

The final review included two RCTs^21^, ^22^ (*n* = 2570, including 1288 receiving mild or moderate potency TCS), two cohort^23^, ^24^ (all participants received some form of TCS *n* = 148) and three case‐control studies (cases *n* = 10 322, controls *n* = 12 201)^25^, ^26^, ^27^ which reported on adverse effects. Identified studies are summarised in Figures 1 and 2 and details of included studies are in Tables 1 and 2.

**TABLE 1:** Characteristics of included studies.

Study name	Design	Number of participants	Population	Exposure	Outcomes	Study length
Andersen 2019^25^	Case control	18 675	Adults, all severities eczema (patients with secondary care dermatology referral excluded), U.K. Primary care CPRD database	Prescription recorded for TCS versus no prescription recorded for TCS (potency and short/long TCS use subgroup analysis)	Type 2 diabetes	Variable, minimum 4 years prospective follow‐up
Arana unpublished^26^	Case control	3383	Adults and children, all severities eczema, U.S.A. Primary and secondary care PharMetrics insurance claims database	Prescription recorded for TCS (but not TCI) versus no prescription recorded for TCS or TCI	Lymphoma	Variable, ≥6 months enrolment required for inclusion
Arellano 2009^27^	Case control	465	Adults and children, all severities, U.K. Primary care THIN database	Prescription recorded for TCS versus no prescription recorded for TCS (or TCI) results reported in high and low potency subgroups.	Lymphoma	Variable, ≥6 months enrolment required for inclusion
Patel 1998^23^	Cohort	77	Children, mild to severely inflamed atopic dermatitis, recruited from U.K. secondary care	Mild potency TCS versus moderate potency TCS	Growth abnormalities	Two years
Sigurgeirsson 2015^22^	RCT	2418	Children, IGA 2‐3 (mild‐moderate), multiple countries	Low or moderate potency TCS (investigator's discretion) used at first signs/symptoms of eczema until eczema clearance or according to manufacturer's instructions and reinitiated at the occurrence of first signs and symptoms of ad flares versus pimecrolimus (PIM) 1% cream twice daily used in the same manner. In the TCI group if a patient experienced a flare TCI was stopped and TCS was used.	Skin thinning	Five years
					Lymphoma	
					Growth abnormalities	
				N.B.: only a third of patients in the PIM [TCI] group did not use any TCS (36%). Median number of days TCS used in the PIM [TCI] group was 7 (Q1:0 Q3: 49 days). Median number of days used in the TCS only group was 178 (Q1:77, Q3:396 days) over the study period.	Non‐skin infections	
					Impaired vaccine response	
					Non‐lymphoma malignancies	
Salava 2021^21^	RCT	152	Children, moderate to severe eczema, Finland	Hydrocortisone acetate 1% cream with hydrocortisone butyrate 0.1% cream (mild/moderate respectively, as classified by the study authors) if needed twice daily for 3–7 days or until clearance versus tacrolimus 0.03% ointment with tacrolimus 0.1% ointment if needed twice daily for 3–7 days or until clearance, then twice weekly	Lymphoma, growth abnormalities, non‐skin infections, impaired vaccine response, non‐lymphoma malignancies, signs of adrenal insufficiency	Three years
van Velsen 2012^24^	Cohort	71	Adults, moderate to severe ad, recruited from secondary care in the Netherlands	<75 g per month TCS (from mild to super potent) versus ≥ 75 g per month TCS (from mild to super potent)	Reduction in bone mineral density	Two years

Abbreviations: AD, Atopic Dermatitis; CPRD, Clinical Pratice Research Datalink; IGA, Investigator's Global Assessment; RCT, Randomised Controlled Trial; TCS, Topical Corticosteroid; TCI, Topical Calcineurin Inhibitor.

**TABLE 2:** Summary of findings: topical corticosteroids (TCS) compared to any other topical therapies for eczema.

Outcomes	Impact	No. participants (studies)	Certainty of the evidence (GRADE)
Skin thinning follow‐up: 5 years	1 case of atrophy with mild or moderate TCS (*n* = 1213); no cases with TCI plus TCS for flares (*n* = 1205)	2418 (1 RCT)^22^	⊕⊕⊕⊝
			Moderate^1^
Type 2 diabetes follow‐up: ≥4 years	TCS (all potencies) was associated with an increased risk of new onset type 2 diabetes: OR = 1.27 (95% CI: 1.19–1.36)	9558 cases, 9117 controls (1 observational study)^25^	⊕⊝⊝⊝
			Very low^2,3^
Lymphoma			
Follow‐up: 3–5 years	No cases of lymphoma with mild or moderate potency TCS (*n* = 1288) or TCI ± TCS for flares (*n* = 1282)	2570 (2 RCTs)^21^, ^22^	⊕⊕⊕⊝
			Moderate^4^
Follow‐up: ≥6 months	Both high and low potency TCS were associated with an increased risk of lymphoma in one study with 465 participants, with adjusted OR = 4.93 (95% CI: 2.28–10.63) and 3.07 (95% CI: 1.55–6.06), respectively. Another study with 3383 participants reported no association, with adjusted OR = 0.90 (95% CI: 0.75–1.07).	3848 (2 observational studies)^26^, ^27^	⊕⊝⊝⊝
			Very low^5^
Growth abnormalities			
Follow‐up: 3–5 years	No difference in height and weight between those treated with mild or moderate potency TCS (*n* = 1288) and those treated with TCI ± TCS for flares (*n* = 1282)	2570 (2 RCTs)^21^, ^22^	⊕⊕⊕⊝
			Moderate^6^
Follow‐up: 2 years	No difference in height, height velocity, or delay in bone age between those treated with moderate potency (*n* = 39) TCS and those treated with mild TCS (*n* = 38)^7^	77 (1 observational study)^23^	⊕⊝⊝⊝
			Very low^8^
Reduction in bone mineral density follow‐up: 2 years	No clinically significant difference in bone mineral density between those using ≥75 g TCS (all potencies) (*n* = 34) and those using <75 g per month (*n* = 37)^9^	71 (1 observational study)^24^	⊕⊝⊝⊝
			Very low^10^
Clinical signs of adrenal insufficiency follow‐up: 3 years	No cases of clinical signs of adrenal insufficiency with mild or moderate TCS (*n* = 75) or TCI (*n* = 77)	152 (1 RCT)^21^	⊕⊕⊕⊝
			Moderate^11^
Non‐skin infections follow‐up: 3–5 years	No difference in non‐skin infections between those treated with mild or moderate TCS (*n* = 1288) and those treated with TCI ± TCS for flares (*n* = 1282)	2570 (2 RCTs)^21^, ^22^	⊕⊕⊕⊝
			Moderate^6^
Impaired vaccine response follow‐up: 3–5 years	No difference in vaccine response between those treated with mild or moderate potency TCS (*n* = 1288) and those treated with TCI ± TCS for flares (*n* = 1282)	2570 (2 RCTs)^21^, ^22^	⊕⊕⊕⊝
			Moderate^6^
Non‐lymphoma malignancies follow‐up: 3–5 years	No cases of cutaneous malignancies in those treated with mild or moderate potency TCS (*n* = 1288) and those treated with TCI ± TCS for flares (*n* = 1282)	2570 (2 RCTs)^21^, ^22^	⊕⊕⊕⊝ Moderate^4^
	2 cases of internal malignancies in those treated with TCS (*n* = 1288; acute lymphocytic leukaemia and ependymoma); 1 case with TCI ± TCS for flares (*n* = 1282; a benign pilomatrixoma)		

*Note*: Patient or population: eczema. Setting: hospital and community settings in predominantly high‐income countries. Intervention: topical corticosteroids. Comparison: any other topical therapies (including topical corticosteroids at a reduced potency or volume where indicated).

Abbreviations: CI, Confidence Interval; OR, odds ratio; RCT, Randomised Controlled Trial; TCI, Topical Calcineurin Inhibitor; TCS, Topical Corticosteroid.

**GRADE Working Group grades of evidence**

**High certainty:** we are very confident that the true effect lies close to that of the estimate of the effect.

**Moderate certainty:** we are moderately confident in the effect estimate: the true effect is likely to be close to the estimate of the effect, but there is a possibility that it is substantially different.

**Low certainty:** our confidence in the effect estimate is limited: the true effect may be substantially different from the estimate of the effect.

**Very low certainty:** we have very little confidence in the effect estimate: the true effect is likely to be substantially different from the estimate of effect.

**Explanations**

1. Downgraded for imprecision due to small number of events. Whilst there were also some concerns on RoB2 assessment due to lack of blinding, this was thought likely to inflate events in the TCS group; as this was not the case we did not downgrade further.

2. Downgraded for risk of bias due to lack of code and prescription record validation, unjustified exclusion of patients referred to dermatology, and insufficient adjustment for relevant confounders (i.e., duration and severity of eczema).

3. Downgraded for inconsistency as systemic corticosteroid use had a lower risk of type 2 diabetes that TCS (OR = 1.18, 95% CI: 1. 09 to 1.28).

4. Downgraded for imprecision due to lack of events. Whilst there were also some concerns on RoB2 assessment due to lack of blinding, this outcome was thought most likely to have been diagnosed by an independent investigator, therefore we did not downgrade further.

5. Downgraded due to inconsistency as one study shows a positive association, but the other does not.

6. Downgraded for risk of bias primarily due to lack of blinding.

7. Please note that in this instance the comparator is a milder TCS rather than vehicle or an alternative topical active comparator with a different mechanism of action.

8. Downgraded twice for risk of bias due to the lack of adjustment for confounding factors resulting in a critical risk judgement along with a serious risk of bias due to missing data.

9. Please note that in this instance the comparator is a smaller volume of the same TCS rather than vehicle or an alternative topical active comparator with a different mechanism of action.

10. Downgraded for risk of bias primarily due to lack of adjustment for relative confounders (i.e., duration and severity of eczema).

11. Downgraded for risk of bias primarily due to lack of blinding as the outcome relies on clinician inspection and may be influenced by knowledge of intervention received.

The two RCTs included children (one with mild to moderate eczema,^22^ the other moderate to severe^21^). One cohort study included only children^23^ (with mild to severe eczema), whilst the other included only adults^24^ (with moderate to severe eczema). Finally all case‐control studies included adults,^25^ with two also including children^26^, ^27^ (all severities).

Contact with study authors is documented in Table S1. A table with reasons for exclusion can be found in Table S2. Risk of bias judgements are available in Table S3.

### 3.2 Adverse effects identified

The seven included studies provided data on nine adverse effects: signs of skin thinning,^22^ type 2 diabetes,^25^ lymphoma,^21^, ^22^, ^26^, ^27^ growth abnormalities,^21^, ^22^, ^23^ reduction in bone mineral density (BMD),^24^ clinical signs of adrenal insufficiency,^21^ non‐skin infections,^21^, ^22^ impaired vaccine response^21^, ^22^ and other non‐lymphoma malignancies.^21^, ^22^

### 3.3 Adverse effects for which no studies were identified

We found no studies reporting TSW, eye problems and mental health issues. No studies reported data on ageing, wrinkling, changes in skin colour, worsening or induction of acne and sensitisation.

### 3.4 Length of included studies

The longest study in terms of duration of follow‐up was a 5‐year RCT by Sigurgeirsson *et al.* ^22^ However, the large database case‐control studies (reporting on type 2 diabetes and lymphoma) are likely to have included longer follow‐up as the length of follow‐up usually depends on the length of time a patient contributes data to the database (the study examining type 2 diabetes had a minimum length of follow‐up of 4 years^25^ and the lymphoma database studies a minimum of 6 months with a maximum of 14 years).^26^, ^27^

### 3.5 Data on identified adverse effects

Additional data for each adverse effect can be found in Table S4. The master data set is available in Table S5.

#### 3.5.1 Local adverse effects

##### Signs of skin thinning

One RCT^22^ reported information on the risk of skin thinning associated with TCS use (Table 2).

The RCT found only one episode of skin atrophy in 1213 patients treated with TCS. In this study, children with mild to moderate eczema were randomised to use low to moderate potency TCS or topical calcineurin inhibitors (TCI) to treat flares over a period of 5 years (moderate certainty of evidence).

#### 3.5.2 Systemic adverse effects

##### Type 2 diabetes

One case‐control study^25^ with 9558 cases and 9117 controls looked at the risk of type 2 diabetes associated with TCS use (Table 2). This study used routinely collected healthcare data for adults from the U.K. Clinical Pratice Research Datalink primary care database. There was a slightly increased risk of type 2 diabetes for people using TCS (any potency) compared to people without TCS‐use, adjusted OR adjusted OR 1.27 (95% CI 1.19–1.36) (evidence was assessed as “very low” certainty). See Table S5 for further details of additional subgroup analyses by potency and length of use.

##### Lymphoma

Two RCTs^21^, ^22^ and two case‐control studies^26^, ^27^ reported on the risk of lymphoma associated with TCS. One case‐control study^26^ had previously been published partially elsewhere,^28^ however we obtained further complete data from the author.

We found conflicting results (three studies found no increased risk^21^, ^22^, ^26^ and one study suggested increased risk^27^). No cases of new cases of lymphoma were identified in either of the RCTs (*n* = 2418 and *n* = 152) which were collectively assessed as “moderate” certainty evidence (Table 2). Both RCTs compared risk associated with mild or moderate TCS versus risk with TCI. Similarly, in one of the case‐control studies (with 670 cases and 2713 controls)^26^ using data from a large U.S. database, no association between TCS and lymphoma was found, adjusted OR 95%CI 0.90 (0.75–1.07) (Table 2). See Table S5 for further details of additional subgroup analyses by age and lymphoma subtype.

However, in one of the case‐control studies (Table 2), which used UK‐derived THIN primary care data,^27^ a positive association with TCS use and lymphoma risk was reported (high potency vs. no TCS adjusted OR 95%CI 4.93 (2.28–10.63) with 94 cases and 371 controls, low potency versus no TCS OR 95% CI adjusted 3.07 (1.55–6.06)) with 94 cases and 371 controls. The evidence from the case‐control studies was assessed as “very low” certainty.

##### Growth abnormalities

Two RCTs^21^, ^22^ and one cohort study^23^ assessed the risk of growth abnormalities associated with the use of TCS use in children (Table 2). No studies identified any differences in growth between groups.

Both RCTs compared growth in children using mild or moderate TCS versus growth in children using TCI. The RCTS included a three‐year study with 152 participants^21^ and one much larger (TCS *n* = 1213, TCI *n* = 1205) and longer five‐year RCT^22^ (evidence assessed as “moderate” certainty).

The cohort study had a follow‐up time of 2 years. The study was small, including 77 patients,^23^ and compared growth measurements in patients using mild versus moderate TCS. The observational evidence from this study was assessed as “very low” certainty.

##### Reduction in BMD

One cohort study,^24^ which included 71 adults with moderate to severe eczema, evaluated the risk of reduction in BMD in two groups with different levels of exposure to TCS of any potency (<75 g and ≥75 g TCS use) (Table 2) and measured the percentage change in BMD using dual energy x‐ray absorptiometry at baseline and after two years. A clinically significant difference in hip or spine BMD was not found between patients using <75 g and ≥75 g TCS per month (evidence was assessed as “very low” certainty).

##### Clinical signs of adrenal insufficiency

An RCT,^21^ 3 years in length, reported the number of patients that had clinical signs of insufficiency and reported no events. The participants included 75 children (aged 1–3 years) who used TCS and 77 children who used TCI (Table 2). The trial involved participants applying TCS or TCI if needed to clear a flare. The TCS used was hydrocortisone acetate 1% cream however hydrocortisone butyrate 0.1% cream (mild/moderate potency as defined by the study authors) could be applied (assessed as “moderate” certainty evidence).

##### Other adverse effects (non‐skin infections, impaired vaccine response and non‐lymphoma malignancies)

Two RCTs^21^, ^22^ (three and five years long) reported on non‐skin infections, impaired vaccine response and other malignancies (*n* = 2418 and *n* = 152). No significant differences were found when mild/moderate TCS were compared to TCI with regards to non‐skin infections (“moderate” certainty evidence), impaired vaccine response (“moderate” certainty evidence) and non‐lymphoma malignancies (“moderate” certainty evidence) (Table 2).

## 4 DISCUSSION

### 4.1 Main findings

This systematic review of studies that examined longer‐term effects of TCS has identified seven studies evaluating a range of local and systemic adverse effects. This review found no clear evidence to suggest safety concerns of TCS when used over longer periods, but some very low‐certainty observational studies found potential associations that warrant further investigation.

Data from the PETITE study,^22^ in which mild to moderate TCS were studied, is reassuring in that only one episode of skin thinning was reported within a large patient population who used TCS over 5 years. However, this study only included children and so we still do not have information regarding the safety of TCS when used in older populations.

No association between TCS and growth abnormalities was found in all three studies of children using mainly mild/moderate potency TCS. Two of these studies provided evidence that was assessed as “moderate” certainty evidence. Furthermore, it is difficult to attribute growth impairment to TCS use as severe eczema itself can be a cause of growth impairment in children. One study has suggested that faltering growth can begin as early as in utero and precede development of atopic eczema.^29^ Collectively, these data and reasonings should be reassuring to healthcare professionals and patients when considering the balance of risks between leaving eczema untreated and using TCS. Of note, none of the studies included a non‐treatment group.^21^, ^22^, ^23^ This means that if the active control treatment also caused growth abnormalities, any risk would not be identified.

One study in adults found no association between TCS use and reduction in BMD. Though this was a small study, precise measures of BMD were used so the results are useful. However, a key problem with this study was that patients had been using TCS for many years before the study, so duration of exposure was hard to establish. Future studies could include a cohort of patients newly starting TCS medication.

A small, positive association was found between TCS and type 2 diabetes in a large observational study, but this finding is difficult to interpret due to methodological limitations and inconsistencies within the results. There was a smaller association found between systemic corticosteroids and type 2 diabetes than was found for topical treatment, which appears to be counter‐intuitive and may suggest that there is residual confounding. Where possible, more refined measures of eczema severity and duration of eczema should be used to allow adjustment for the potential confounding effect of these variables and consideration of more advanced design and analytical approaches to address confounding by indication or severity.

Three out of the four studies reporting on the risk of lymphoma found no association between lymphoma and TCS. Of note the two RCTs (contributing moderate certainty evidence), did not identify any cases of lymphoma. Although a small risk of lymphoma cannot be completely excluded, it is possible that the association that was found in the one case control study was due to either residual confounding (as eczema itself is associated with a small risk of lymphoma^7^) or due to surveillance bias.

### 4.2 Comparisons with other reviews

An umbrella review of systematic reviews looked at the safety of TCS.^11^ This review highlighted that “long‐term safety data were limited”. We identified three further studies not included in the umbrella review, including one RCT, one case‐control, and one cohort study. The 3‐year RCT contributed data on the effect of TCS on growth abnormalities, signs of clinical adrenal insufficiency, effects on the immune system, lymphoma and non‐lymphoma malignancies (moderate certainty evidence).

### 4.3 Strength and limitations

This review included studies with follow‐up of greater than 12 months to assess long‐term adverse effects of TCS. Although some of the observational database studies included longer follow‐up, the longest RCT was 5 years in duration. Longer‐term studies are needed as treatment with intermittent use of TCS for some people with eczema might be lifelong. In general, adverse effects were poorly and inconsistently reported. There was virtually no information regarding the resolution and impact of the adverse effects. There was no information on eye symptoms, mental health impacts, TSW or local symptoms (other than those reported signs of skin thinning). Furthermore, the studies which involve the use of large primary care databases rely upon the clinician, who is providing routine care, to identify that the patient had suffered a particular adverse effect and to code for this in their electronic record. Therefore, there is likely to be under‐reporting of adverse effects in the clinical record.

It is also worth pointing out that even high quality RCTs may not capture rare but important long‐term adverse events.

### 4.4 Recommendations for practice

Despite the fact there are new emerging treatments for eczema, TCS are likely to remain the mainstay of treatment for most people with eczema who are treated in primary care due to their effectiveness in controlling inflammation and relative safety record when used intermittently to treat eczema flares.

This systematic review provides a comprehensive and critical description of all the available safety data from RCTs, cohort studies and case‐control studies when TCS were used for more than a year. This review should inform balanced discussions between people with eczema (especially those who are nervous of using TCS) and healthcare professionals, and can be used in conjunction with other self‐education resources such as the www.eczemacareonline.org.uk website, which provides accessible evidence‐based self‐management support for people with eczema.

### 4.5 Recommendations for future research

Longer term studies of TCS safety are required which look at adverse effects over the course of decades to reflect life‐long usage.

There are several adverse effects where we identified no data, for example, in the case of TSW. A recent paper called for observational studies of TSW and this review reinforces this research gap.^30^ Finally, the development of a core outcome set of adverse effects associated with TCS use would standardise the recording of adverse effects for all skin conditions, improve the quality of future research and allow meta‐analysis of adverse effect data.

## 5 CONCLUSION

Taken overall, the body of evidence provided some reassurance that TCS used intermittently for the management of eczema is safe over periods of up to 5 years. Gaps remain in our understanding of the life‐long effects of TCS use and higher quality studies which address all relevant safety outcomes and include longer follow‐up are needed.

## CONFLICT OF INTEREST STATEMENT

Jane Harvey, Stephanie J. Lax, have declared that they have no conflict of interest. Alison Lowe has worked for AbbVie and has been sponsored to attend conference and educational events by AbbVie and Eli‐Lilly. Sandra Lawton is funded by an honorarium (Thorton and Ross – lecture). Sandra Lawton took part in a Podcast transitioning young people with Eczema (funded by Abbvie). Sinead M. Langan is funded by a Wellcome Senior Clinical fellowship. Sinead M. Langan is an investigator on the European Union Horizon 2020‐funded BIOMAP Consortium (http://www.biomap‐imi.eu/), but not in receipt of industry funding. Miriam Santer, Sandra Lawton, Sinead M. Langan, Amanda Roberts, Beth Stuart, Hywel C. Williams and Kim S. Thomas are ECO co‐applicants. HCW and KST are members of the Harmonising Outcomes Measures for Eczema Executive Group.

## AUTHOR CONTRIBUTION

**Jane Harvey**: Data curation (lead); formal analysis (lead); investigation (equal); methodology (lead); project administration (lead); writing – original draft (lead); writing – review & editing (equal). **Stephanie J. Lax**: Data curation (lead); formal analysis (lead); investigation (equal); methodology (lead); project administration (lead); writing – original draft (lead); writing – review & editing (equal). **Alison Lowe**: Data curation (equal); formal analysis (equal); investigation (equal); methodology (equal); writing – review & editing (equal). **Miriam Santer**: Conceptualization (equal); funding acquisition (lead); investigation (equal); methodology (equal); writing – review & editing (equal). **Sandra Lawton**: Conceptualization (equal); funding acquisition (equal); investigation (equal); writing – review & editing (equal). **Sinead M. Langan**: Conceptualization (equal); funding acquisition (equal); investigation (equal); methodology (equal); writing – review & editing (equal). **Amanda Roberts**: Conceptualization (equal); funding acquisition (equal); investigation (equal); writing – review & editing (equal). **Beth Stuart**: Conceptualization (equal); funding acquisition (equal); investigation (equal); methodology (equal); writing – review & editing (equal). **Hywel C. Williams**: Conceptualization (equal); funding acquisition (equal); investigation (equal); methodology (equal); writing – review & editing (equal). **Kim S. Thomas**: Conceptualization (equal); funding acquisition (lead); investigation (equal); methodology (equal); supervision (lead); writing – review & editing (equal).

## ETHICS STATEMENT

Not applicable.

## Supporting information

Supplementary Material

Supplementary Material

Table S1

Table S2

Table S3

Table S4

Table S5

## Data Availability

Data available in article supplementary material.
