# Supplementary material for: The long‐term safety of topical corticosteroids in atopic dermatitis: A systematic review
Source: Skin Health Dis. 2023 Aug 16;3(5):e268. doi: 10.1002/ski2.268 (PMC10549798; doi:10.1002/ski2.268)
Supplement: Supplementary file 1 — Supplementary Material [file SKI2-3-e268-s002.docx]

We originally stated that we required a minimum cohort size of 100 for the observational studies or minimum 100 per arm for the RCTs for inclusion within this review. This was originally a pragmatic decision in order to exclude studies that included very few patients. However, we decided, after full text screening, that this arbitrary decision would mean the exclusion of half of the studies that could contribute useful information, both in terms of results and in terms of describing the methods that could be used in future studies. Therefore, we decided to revisit this decision and amend the minimum cohort size to 50 per cohort or a minimum of 50 per arm in the RCTs.

In addition, we stated in our protocol that we would assess observational studies using the ROBINS-I tool (Sterne 2016). However, at the time of writing, the ROBINS-I tool had not yet been adapted for use in assessing case-control studies. Therefore, we used the Newcastle Ottawa Scale to assess the risk of bias within case-control studies.

We included narrative as well as numerical data
