# Supplementary material for: The long‐term safety of topical corticosteroids in atopic dermatitis: A systematic review
Source: Skin Health Dis. 2023 Aug 16;3(5):e268. doi: 10.1002/ski2.268 (PMC10549798; doi:10.1002/ski2.268)

**Details of searches 09/12/2021**

These searches utilised the Ovid MEDLINE and Embase observational studies specialist filter in conjunction with terms for atopic dermatitis and topical corticosteroids (as shown below)

<https://sites.google.com/a/york.ac.uk/issg-search-filters-resource/home/observational-studies>

<https://tools.ovid.com/ovidtools/expertsearches.html>

**Ovid MEDLINE(R) ALL 1946 to December 08, 2021**

1. Epidemiologic studies/
2. exp case control studies/
3. exp cohort studies/
4. Case control.tw.
5. (cohort adj (study or studies)).tw.
6. Cohort analy$.tw.
7. (Follow up adj (study or studies)).tw.
8. (observational adj (study or studies)).tw.
9. Longitudinal.tw.
10. Retrospective.tw.
11. Cross sectional.tw.
12. Cross-sectional studies/
13. or/1-12
14. (eczema or dermatitis or neurodermatitis).mp. or exp Dermatitis, Atopic/
15. (alclometasone or amcinonide or beclometasone or beclomethasone or betamethasone or budesonide or clobetasol or clobetasone or clocortolone or deprodone or desonide or desoximetasone or dexamethasone or dichlorisone or diflorasone or diflucortolone or difluprednate or fluclorolone or flucloronide or fludrocortisone or fludroxycortide or flumetasone or flumethasone or fluocinolone or fluocinonide or fluocortin or fluocortolone or fluorometholone or fluprednidene or flurandrenolide or flurandrenolone or fluticasone or halcinonide or halobetasol or halometasone or hydrocortisone or masipredone or mazipredone or methylprednisolone or mometasone or prednicarbat* or prednisolone or prednisone or triamcinolone or ulobetasol or (topical$ adj3 corticosteroid$) or (topical$ adj3 steroid$) or (topical$ adj3 corticoid$) or (topical$ adj3 glucocorticoid$)).mp.
16. 13 and 14 and 15


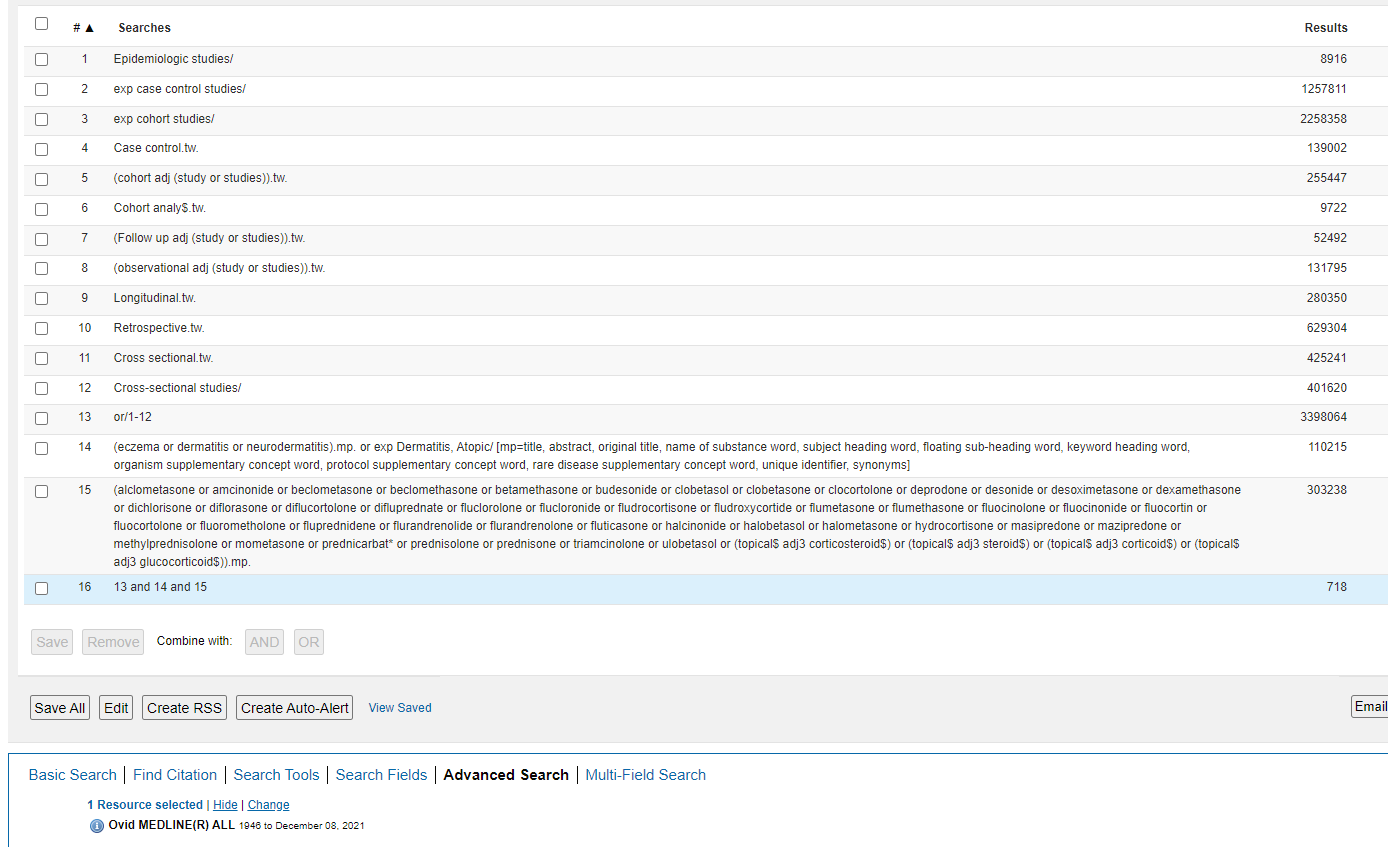


**OVID**Embase **1974 to 2021 December 08**

1. Clinical study/
2. Case control study/
3. Family study/
4. Longitudinal study/
5. Retrospective study/
6. Prospective study/
7. Randomized controlled trials/
8. 6 not 7
9. Cohort analysis/
10. (Cohort adj (study or studies)).mp.
11. (Case control adj (study or studies)).tw.
12. (follow up adj (study or studies)).tw.
13. (observational adj (study or studies)).tw.
14. (epidemiologic$ adj (study or studies)).tw.
15. (cross sectional adj (study or studies)).tw.
16. or/1-5,8-15
17. (alclometasone or amcinonide or beclometasone or beclomethasone or betamethasone or budesonide or clobetasol or clobetasone or clocortolone or deprodone or desonide or desoximetasone or dexamethasone or dichlorisone or diflorasone or diflucortolone or difluprednate or fluclorolone or flucloronide or fludrocortisone or fludroxycortide or flumetasone or flumethasone or fluocinolone or fluocinonide or fluocortin or fluocortolone or fluorometholone or fluprednidene or flurandrenolide or flurandrenolone or fluticasone or halcinonide or halobetasol or halometasone or hydrocortisone or masipredone or mazipredone or methylprednisolone or mometasone or prednicarbat* or prednisolone or prednisone or triamcinolone or ulobetasol or (topical$ adj3 corticosteroid$) or (topical$ adj3 steroid$) or (topical$ adj3 corticoid$) or (topical$ adj3 glucocorticoid$)).mp.
18. (eczema or dermatitis or neurodermatitis).mp.
19. atopic dermatitis/
20. 18 or 19
21. 16 and 17 and 20


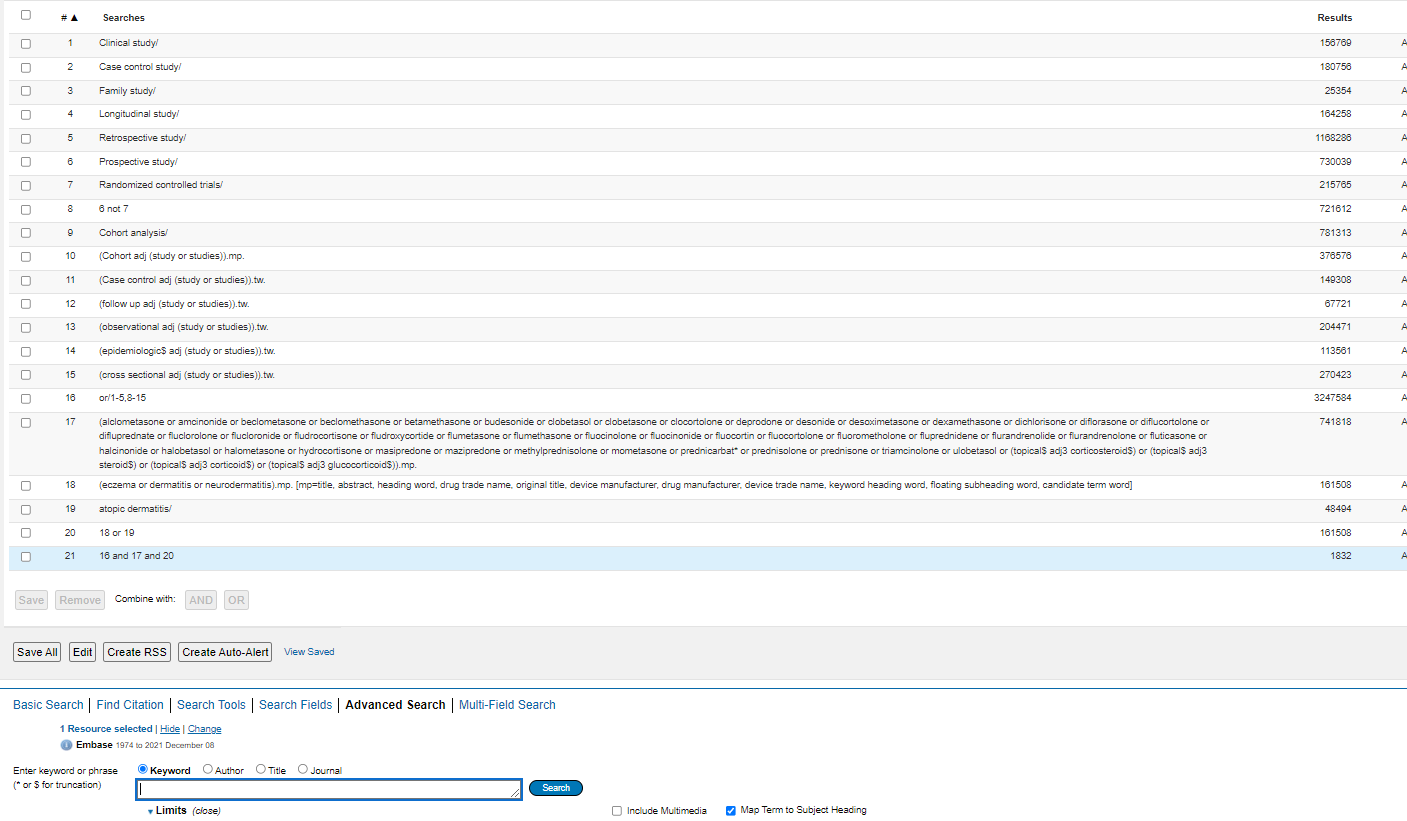

Supplement: Supplementary file 2 — Supplementary Material [file SKI2-3-e268-s001.docx]
