# Supplementary material for: The long‐term safety of topical corticosteroids in atopic dermatitis: A systematic review
Source: Skin Health Dis. 2023 Aug 16;3(5):e268. doi: 10.1002/ski2.268 (PMC10549798; doi:10.1002/ski2.268)
Supplement: Supplementary file 3 — Table S1 [file SKI2-3-e268-s005.docx]

| **Study name** | **Contact** | **Email address** | **Date of contact** | **Reason for contact** | **Outcome** |
| --- | --- | --- | --- | --- | --- |
| To what extent are topical tacrolimus or pimecrolimus associated with increased risk of skin cancer and lymphoma? Long term results from Joelle study. Pharmacoepidemiology and Drug Safety - Volume 29, Issue 0, pp. 568-569 - published 2020-01-01 | Alejandro Arana | aarana@rti.org | 9/3/2022 | Clarify if any results are for AD only cohort | Authors reply: “I am sorry to tell you that there is no way to obtain results for users of TCS in patients with AD. JOELLE was conducted in electronic health record databases from UK, Denmark, Sweden and The Netherlands. Some of the were hospital records and others general practitioner records. The sensitivity of AD from hospital records was very low. We decided to include TCS users if they had an AD diagnosis, or if they got more than one prescription even if they had no diagnoses of AD. We did not analyse the data separately and we cannot identify any results from these studies that are taken from patients with an AD diagnosis only.” |
| “ADDRESSJ study” (UMIN Clinical TrialRegistration:UMIN000022623) | Norito Katoh | nkatoh@koto.kpu-m.ac.jp | 9/3/2022 | Ask if any AE data collected within the  ADDRESSJ study | "Address-J" does not collect any data on the side effects of topical corticosteroids. |
| Association Between Topical Calcineurin Inhibitor Use and Keratinocyte Carcinoma Risk Among Adults With Atopic Dermatitis. JAMA Dermatol. 2020 Oct 1;156(10):1066-1073. doi: 10.1001/jamadermatol.2020.2240 | MM Asgari | pores@mgh.harvard.edu | 9/3/2022 | Check whether the cohort only includes AD patients | No reply |
| Association Between Topical Calcineurin Inhibitor Use and Keratinocyte Carcinoma Risk Among Adults With Atopic Dermatitis. JAMA Dermatol. 2020 Oct 1;156(10):1066-1073. doi: 10.1001/jamadermatol.2020.2240 | MM Asgari | MASGARI@PARTNERS.ORG | 10/6/2022 | Resent email to new address and asked for SA for AD code only | No reply |
| Eczema is a risk factor for incident attention-deficit/hyperactivity disorder British Journal of Dermatology - Volume 167, Issue 2, pp. e4 - published 2012-01-01 | Jochen Schmitt | jochen.schmitt@uniklinikum-dresden.de | 10/3/2022 | Check whether the cohort only includes AD patients | “The talk was a summary of a series of epidemiological papers on the comorbidity of atopic eczema and ADHD. The safety of TCS was not investigated in this. If you search in Medline for Romanos M and Schmitt J you will find the papers easily” |
| Risk of lymphoma following exposure to calcineurin inhibitors and topical steroids in patients with atopic dermatitis. J Invest Dermatol. 2007 Apr;127(4):808-16. doi: 10.1038/sj.jid.5700622. Epub 2006 Nov 9.  Incidence of lymphoma in a large population of patients with atopic dermatitis, some treated with topical calcineurin inhibitors or topical corticosteroids  Allergy: European Journal of Allergy and Clinical Immunology - Volume 67, Issue 0, pp. 15 - published 2012-01-01 | Alejandro Arana | aarana@rti.org | 24/03/2022 | Clarification that the study included AD patients also with the following question:  “we have identified 3 abstracts with similar data, one of which I have attached. Please can you tell me the relationship between the patients in Arellano 2007 and Arana 2012, as we don't want to double count any patients. | “We included  691.8 (dermatitis other  atopic) and 691 (dermatitis atopic) but not 691.0 (rash diaper or napkin) or any other 692.”  “They are the same study in the Pharmetrics database.” “I am attaching the submitted manuscript in case is helpful. Please take into account that it has been peer reviewed and rejected (not new [data])” |
