# Supplementary material for: The long‐term safety of topical corticosteroids in atopic dermatitis: A systematic review
Source: Skin Health Dis. 2023 Aug 16;3(5):e268. doi: 10.1002/ski2.268 (PMC10549798; doi:10.1002/ski2.268)
Supplement: Supplementary file 4 — Table S2 [file SKI2-3-e268-s004.docx]

| **Excluded study** | **Authors** | **Source** | **Reason for exclusion** |
| --- | --- | --- | --- |
| To what extent are topical tacrolimus or pimecrolimus associated with increased risk of skin cancer and lymphoma? Long term results from Joelle study. Pharmacoepidemiology and Drug Safety - Volume 29, Issue 0, pp. 568-569 - published 2020-01-01 | Arana, Alej and ro and Gutierrez, Lia and Perez-Gutthann, Susana and Pottegard, Anton and Lund, Lars Christian and Hallas, Jesper and Kuiper, Josephina G. and Houben, Eline and Crellin, Elizabeth and Booth, Helen and Dedman, Daniel and Reutfors, Johan and Gembert, Karin and Kieler, Helle and Schmitt-Egenolf, Marcus and Calingaert, Brian and Kaye, James A. and Rothman, Kenneth J. | Original search | Population included non-AD patients (see contact with author Table) |
| Association Between Topical Calcineurin Inhibitor Use and Keratinocyte Carcinoma Risk Among Adults With Atopic Dermatitis. JAMA Dermatol. 2020 Oct 1;156(10):1066-1073. doi: 10.1001/jamadermatol.2020.2240 | Asgari MM, Tsai AL, Avalos L, Sokil M, Quesenberry CP Jr. | Original search | Unsure if population exclusively included patients with atopic dermatitis (AD) attempted to contact author no reply (see contact with author Table) |
| A cohort study on the risk of lymphoma and skin cancer in users of topical tacrolimus, pimecrolimus, and corticosteroids (Joint European Longitudinal Lymphoma and Skin Cancer Evaluation - JOELLE study) Clinical epidemiology - Volume 10, Issue 0, pp. 299-310 - published 2018-01-01 | Castellsague, Jordi; Kuiper, Josephina G.; Pottegard, Anton; Anveden Berglind, Ingegard; Dedman, Daniel; Gutierrez, Lia; Calingaert, Brian; van Herk-Sukel, Myrthe Pp; Hallas, Jesper; Sundstrom, Anders; Gallagher, Arlene M.; Kaye, James A.; Pardo, Carolina; Rothman, Kenneth J.; Perez-Gutthann, Susana. | Original search | Population included non-AD patients (see contact with author Table) |
| Clinical dose and adverse effects of topical steroids in daily management of atopic dermatitis The British journal of dermatology - Volume 148, Issue 1, pp. 128-33 - published 2003-01-01 | Furue, M. and Terao, H. and Rikihisa, W. and Urabe, K. and Kinukawa, N. and Nose, Y. and Koga, T. | Original search | the duration of follow up is only 6 months – inadequate study duration |
| Intermittent dosing of fluticasone propionate cream for reducing the risk of relapse in atopic dermatitis patients. Br J Dermatol. 2002 Sep;147(3):528-37. doi: 10.1046/j.1365-2133.2002.05006.x. PMID: 12207596. | Hanifin, J. and Gupta, A. K. and Rajagopalan, R. | Original search | Inadequate study duration |
| Evaluation of the atrophogenic potential of topical corticosteroid in dermatology paediatric patients Pediatr Dermatol. 2011 Jul-Aug;28(4):393-6. doi: 10.1111/j.1525-1470.2011.01445.x. Epub 2011 Apr 20. PMID: 21507057. | Hong, E. and Fischer, G. and Smith, S. D. | Original search | Inadequate study duration |
| Allergic disease, corticosteroid use, and risk of Hodgkin lymphoma: A United Kingdom nationwide case-control study. J Allergy Clin Immunol. 2020 Mar;145(3):868-876. doi: 10.1016/j.jaci.2019.10.033. | Rafiq M, Hayward A, Warren-Gash C, Denaxas S, Gonzalez-Izquierdo A, Lyratzopoulos G, Thomas S. | Handsearch of included papers | Risk of Hodgkin lymphoma for eczema OR topical corticosteroid, but does not include exposure to both |
| Eczema is a risk factor for incident attention-deficit/hyperactivity disorder British Journal of Dermatology - Volume 167, Issue 2, pp. e4 - published 2012-01-01 | Schmitt, J. | Original search | Did not look at the safety of topical corticosteroids |
| Topical treatments with pimecrolimus, tacrolimus and medium- to high-potency corticosteroids, and risk of lymphoma Dermatology 2009;219:7–21. | Schneeweiss, Sebastian and Doherty, Mike and Zhu, Shao and Funch, Donnie and Seeger, John D. and Fern and ez-Vidaurre, Carlos and Schlienger, Raymond G. | Original search | Eczema data not separated - Population included non-AD patients |
| Retrospective markers of paediatric atopic dermatitis persistence after hospital diagnosis: A nationwide cohort study Clin Exp Allergy. 2019 Nov;49(11):1455-1463. doi: 10.1111/cea.13487. Epub 2019 Sep 11. PMID: 31464039. | Thyssen JP, Corn G, Wohlfahrt J, Melbye M, Bager P. | Original search | No safety data |
| Efficacy and safety of pimecrolimus cream in the long-term management of atopic dermatitis in children Pediatrics. 2002 Jul;110(1 Pt 1): e2. doi: 10.1542/peds.110.1.e2. PMID: 12093983. | Wahn, Ulrich and Bos, Jan D. and Goodfield, Mark and Caputo, Ruggero and Papp, Kim and Manjra, Ahmed and Dobozy, Attila and Paul, Carle and Molloy, Stephen and Hultsch, Thomas and Graeber, Michael and Cherill, Robert and de Prost, Yves and Flare Reduction in Eczema with Elidel Multicenter Investigator Study, Group. | Original search | Inadequate study duration |

*AD – Atopic dermatitis*
