# Supplementary material for: The long‐term safety of topical corticosteroids in atopic dermatitis: A systematic review
Source: Skin Health Dis. 2023 Aug 16;3(5):e268. doi: 10.1002/ski2.268 (PMC10549798; doi:10.1002/ski2.268)
Supplement: Supplementary file 6 — Table S4 [file SKI2-3-e268-s003.docx]

Table 1 Results from studies reporting on the risk of local adverse effects with topical corticosteroid use

| **Study**  **Study Type** | **Population size (N,) follow-up time (years)** | **Main reported results** |
| --- | --- | --- |
| **Signs of skin thinning** | | |
| Sigurgeirsson 2015  (Randomised Controlled Trial) | TCS n=1213  TCI plus TCS for flares n=1205  Follow-up time up to 5 years | Number of participants with skin atrophy   - TCS n= 1/1213 - TCI plus TCS for flares n= 0/1205   Number of participants with Telangiectasia (sign of skin thinning)   - TCS n= 2/1213 - TCI plus TCS for flares n= NOT REPORTED |

TCS -Topical corticosteroid TCI – Topical calcineurin inhibitor

Table 2 Results from studies reporting on the risk of type 2 diabetes with topical corticosteroid use

| **Study**  **Study type** | **Analysis type** | **Proportion of cases (with type 2 diabetes) and controls (without type 2 diabetes) exposed to TCS** | **Main reported results (OR, 95% CI)*** | **Notes** |
| --- | --- | --- | --- | --- |
| Andersen 2019  (Case-control) | Any TCS use | Cases: 6223/9558 (65.2%)  Controls 5187/9117 (57.1%) | 1.27 (1.19-1.36) | TCS was associated with an increased risk of new onset type 2 diabetes regardless of the different duration and potency categories.  Inconsistency; greater risk for type 2 diabetes was found with topical corticosteroid use than with systemic corticosteroid use (systemic corticosteroid adjusted OR 1.18, (95% CI 1. 09-1.28). Also, no dose response seen across the potency or length of exposure subgroup analyses.  NB: everyone other than people with very mild AE are likely to have used TCS at some point, so it is unclear how representative the "no TCS" participants are as a comparison group |
|  | Former short use of any TCS | Cases: 2428/9558 (25.4%)  Controls: 2283/9117 (25.1%) | 1.17 (1.08-1.27) |  |
|  | Former long use of any TCS | Cases: 288/9558 (3.01%)  Controls: 235/9117 (2.59%) | 1.25 (1.03-1.52) |  |
|  | Current short use of any TCS | Cases :1689/9558 (17.7%)  Controls: 1689/9117 (14.5%) | 1.38 (1.26-1.52) |  |
|  | Current long use of any TCS | Cases: 1818/9558 (19%)  Controls: 1352/9117 (14.9%) | 1.33 (1.21-1.46) |  |
|  | Mild TCS use (any time/duration) | Cases: 1386/9558 (14.5%)  Controls: 1207/9117 (13.3%) | 1.24 (1.12-1.37) |  |
|  | Moderate TCS use (any time/duration) | Cases: 995/9558(10.4%)  Controls: 857/9117 (9.40%) | 1.26 (1.13-1.41) |  |
|  | Potent TCS use (any time/duration) | Cases 3053/9558 (32.0%)  Controls: 2544/9117 (28.0%) | 1.25 (1.16-1.35) |  |
|  | Very potent TCS use (any time/duration) | Cases: 789/9558 (8.3%)  Controls: 580/9117 (6.4%) | 1.43 (1.26-1.62) |  |

CI – confidence interval, OR- odds ratio. TCS -Topical corticosteroid *Unconditional logistic regression, all results reported are adjusted for age, sex, index of multiple deprivation, systemic CS, smoking status, body mass index, inhaled corticosteroids, psoriasis, and eczema. Long term use of TCS was defined as prescriptions in two consecutive years or more, and current use was defined as a prescription less than one year prior to index. No exposure was used as the reference.

Table 3 Results from studies reporting on the risk of lymphoma associated with topical corticosteroid use

| **Study** | **Population size (N,) follow-up time (years)** | **Main reported results** | | **Notes** |
| --- | --- | --- | --- | --- |
|  | | **Narrative results** | |  |
| Sigurgeirsson 2015 (Randomised Controlled Trial) | TCS n=1213  TCI plus TCS for flares n=1205  Follow-up time up to 5 years | To quote: "There were no cases of T-cell lymphoma […] with PIM (TCI) during the study."  "Two malignancies occurred in the TCS group (acute lymphocytic leukemia, ependymoma), and 1 benign tumour was reported in the PIM group (pilomatrixoma)." | |  |
| Salava 2021  (Randomised Controlled Trial) | TCS n=75  TCI n=77  Follow-up time 3 years | Quote: "There were no cutaneous or internal malignancies identified during the 36-month follow-up period in either group." | |  |
|  | | **Proportion exposed to TCS** | **Odds ratios (95% CI)** |  |
| Arellano 2009  (Case control) | high potency vs no TCS  Lymphoma cases n=94 Controls n=371 | Lymphoma cases 37/94 (39%) Controls 89/371 (24%) | Adjusted^a^ 4.93(2.28-10.63) | Inconsistency: observational studies conflict, Arellano 2009 identifies positive association whilst Arana unpublished finds no association.  NB: Although a difference is seen between high and low potency in the Arellano 2009 study, implying a dose response, the CI overlaps reducing the likelihood of an association. |
|  | low potency vs no TCS  Lymphoma cases n=94 Controls n=371 | Lymphoma cases 42/94 (45%) Controls 151/371 (41%) | Adjusted^a^ 3.07 (1.55-6.06) |  |
| Arana unpublished (Case control) | Overall, TCS v no TCS  Lymphoma cases n=670 Controls n=2713 | Lymphoma cases 301/670 (44.9%) Controls 1256/2713= (46.3%) | Unadjusted 0.94 (0.80-1.12) Adjusted^b^. 0.90 (0.75-1.07) |  |
|  | < 20 years old, TCS v no TCS  Lymphoma cases n=158 Controls n=626 | Lymphoma cases 56/158 (35%) Controls 265/626 (42%) | Unadjusted. 0.75 (0.52 - 1.08) Adjusted^b^ 0.72 (0.48-1.06) |  |
|  | T lymphoma subtype, TCS v no TCS  Lymphoma Cases n=96 Controls n=434 | Lymphoma cases 51/96 (53%) Controls 206/434 (47%) | Unadjusted. 1.27 (0.8 – 2.00) Adjusted^b^ 1.16 (0.69-1.93) |  |

CI – confidence interval TCS -Topical corticosteroid TCI – Topical calcineurin inhibitor ^a^Adjusted for sex, age, practice, presence of infectious mononucleosis, asthma diagnosis, oral steroid use, and severity of AD using logistic, fixed effects logistic regression model using backwards elimination process ^b^Matched for age, sex and index date, adjusted for AD index year, region, specialty, presence of infectious mononucleosis, asthma diagnosis, asthma drug use, oral corticosteroids use and severity of AD using conditional logistic regression

Table 4 Results from studies reporting on the risk of growth abnormalities, bone mineral density reduction and clinical signs of adrenal insufficiency associated with topical corticosteroid use

| **Study** | **Population size (N,) follow-up time (years)** | **Main reported results** |
| --- | --- | --- |
| **Growth abnormalities** | | |
| Sigurgeirsson 2015  (Randomised Controlled Trial) | TCS n=1213  TCI plus TCS for flares n=1205  Follow-up time up to 5 years | To quote: "There was no difference in growth rate between the groups."  Growth velocity height (cm)  TCS mean (SD) 114.5 (5.9) n=871  TCI with TCS for flares mean (SD) 114.4 (5.26) n=833  Growth velocity weight (kg)  TCS mean (SD) 21.4 (3.7) n=871  TCI with TCS for flares mean (SD) 21.1 (3.51) n=833 |
| Salava 2021  (Randomised Controlled Trial) | TCS n=75  TCI n=77  Follow-up time up to 3 years | To quote: "Comparisons between the TCS and TAC [TCI] groups showed no significant differences in height (P = 0.60, 95% CI 0.54–0.70) or weight (P = 0.81, 95% CI 0.75–0.87) at baseline or 36 months." |
| Patel 1998  (Cohort) | 80 (77 consented and satisfied the inclusion and exclusion criteria)  Mild TCS n=38  Moderate TCS n=39  Follow-up time up to 2 years | To quote: “Height, height velocity and delay in bone age did not differ between patients treated with mild potency topical glucocorticoids and those treated with moderate potency ones.”  It was unclear whether these narrative results reflected any numerical results which included adjustment for other factors. |
| **Reduction in bone mineral density** | | |
| van Velsen 2012  (Cohort) | <75g TCS use per month n=37  ≥75g TCS use per month n=34  Follow-up time up to 2 years | To quote: "BMD change between baseline and follow-up did not differ significantly between patients using <75 or ≥75 grams of topical corticosteroids per month.” This applied to both unadjusted and adjusted (for age, sex, dairy intake, physical activity, body mass index, and other steroid use) analyses of data from the lumbar spine and hip.  "In the current study the decrease in BMD after 2 years in patients using high (≥75 g per month) amounts of topical corticosteroids was only 0.484% for the total hip, a very small, and clinically not relevant, decrease." This was from the analysis of hip data adjusted for age, sex, dairy intake, physical activity, body mass index, and other steroid use, with SD ± 3.18. |
| **Clinical signs of adrenal insufficiency** | | |
| Salava 2021  (Randomised Controlled Trial) | TCS n=75  TCI n=77  Follow-up time up to 3 years | To quote: “Serum cortisone concentrations were similar in both treatment groups throughout the follow-up with no clinical signs of adrenal insufficiency.” TCS vs TCI at 36 months, p=0.23, with (95%: CI 0.16-0.29).” |

TCS -Topical corticosteroid TCI – Topical calcineurin inhibitor

Table 5 Results from studies reporting on the risk of non-skin infections, impaired vaccine response and non-lymphoma malignancies with topical corticosteroid use

| **Study**  **Type** | **Population size (N,) follow-up time (years)** | **Main reported results** |
| --- | --- | --- |
| **Non-skin infections** | | |
| Salava 2021  (RCT) | TCS n=75  TCI n=77  Follow-up time up to 3 years | To quote: "Group comparisons showed no significant differences in the number of [non-skin-related infections] (P = 0.50, 95% CI 0.44–0.60) or in the number of individual types: respiratory infections (P = 0.27, 95% CI 0.24–0.38), viral rashes (P = 0.66, 95% CI 0.59–0.74), otitis media (P = 0.85, 95% CI 0.84–0.49) and other infections (P = 0.50, 95% CI 0.33–0.49). During follow-up, there were no severe or life-threatening infections observed." |
| Sigurgeirsson 2015  (RCT) | TCS n=1213  TCI plus TCS for flares n=1205  Follow-up time up to 5 years | To quote: “Overall, the type and frequency of AEs including infections were as expected for this patient population." |
| **Impaired vaccine response** | | |
| Salava 2021  (RCT) | TCS n=75  TCI n=77  Follow-up time up to 3 years | To quote: "In the full study cohort, 28 patients (18.4%) had pathological vaccination responses: 15 patients (20.0%) in the TCS group and 13 patients (16.8%) in the TAC [TCI] group, with no significant differences between the two treatment groups (P = 0.62, 95% CI 0.39–0.68)." |
| Sigurgeirsson 2015  (RCT) | TCS n=1213  TCI plus TCS for flares n=1205  Follow-up time up to 5 years | To quote: “Infants treated with PIM or TCSs developed similar and normal antibody titers to common vaccine antigens” |
| **Non-lymphoma malignancies** | | |
| Salava 2021  (RCT) | TCS n=75  TCI n=77  Follow-up time up to 3 years | To quote: "There were no cutaneous or internal malignancies identified during the 36-month follow-up period in either group." |
| Sigurgeirsson 2015  (RCT) | TCS n=1213  TCI plus TCS for flares n=1205  Follow-up time up to 5 years | To quote: "There were no cases of […] skin malignancies with PIM [TCI] during the study." and "Two malignancies occurred in the TCS group (acute lymphocytic leukaemia, ependymoma), and 1 benign tumour was reported in the PIM group (pilomatrixoma)." |

RCT – randomised controlled trial TCS -Topical corticosteroid TCI – Topical calcineurin inhibitor
